# Maize Canopy Temperature Extracted From UAV Thermal and RGB Imagery and Its Application in Water Stress Monitoring

**DOI:** 10.3389/fpls.2019.01270

**Published:** 2019-10-09

**Authors:** Liyuan Zhang, Yaxiao Niu, Huihui Zhang, Wenting Han, Guang Li, Jiandong Tang, Xingshuo Peng

**Affiliations:** ^1^College of Mechanical and Electronic Engineering, Northwest A&F University, Yangling, China; ^2^Key Laboratory of Agricultural Internet of Things, Ministry of Agriculture, Yangling, China; ^3^Water Management and Systems Research Unit, USDA-ARS, Fort Collins, CO, United States; ^4^Institute of Soil and Water Conservation, Northwest A&F University, Yangling, China; ^5^College of Resources and Architectural Engineering, Northwest A&F University, Yangling, China

**Keywords:** stomatal conductance, leaf area index, soil water content, red-green ratio index, Otsu algorithm, nearest neighbor algorithm

## Abstract

To identify drought-tolerant crop cultivars or achieve a balance between water use and yield, accurate measurements of crop water stress are needed. In this study, the canopy temperature (Tc) of maize at the late vegetative stage was extracted from high-resolution red–green–blue (RGB, 1.25 cm) and thermal (7.8 cm) images taken by an unmanned aerial vehicle (UAV). To reduce the number of parameters for crop water stress monitoring, four simple methods that require only Tc were identified: Tc, degrees above non-stress, standard deviation of Tc, and variation coefficient of Tc. The ground-truth temperatures obtained using a handheld infrared thermometer were used to calibrate the temperature obtained from the UAV thermal images and to evaluate the Tc extraction results. Measured leaf stomatal conductance values were used to evaluate the performance of the four Tc-based crop water stress indicators. The results showed a strong correlation between ground-truth Tc and Tc extracted by the red–green ratio index (RGRI)-Otsu method proposed in this study, with a coefficient of determination of 0.94 (*n* = 15) and root mean square error value of 0.7°C. The RGRI-Otsu method was most accurate for estimating temperatures around 32.9°C, but the magnitude of residuals increased above and below this value. This phenomenon may be attributable to changes in canopy cover (leaf curling) under water stress, resulting in changes in the proportion of exposed sunlit soil in UAV thermal orthophotographs. Therefore, to improve the accuracy of maize canopy detection and extraction, optimal methods and better strategies for eliminating mixed pixels are needed. This study demonstrates the potential of using high-resolution UAV RGB images to supplement UAV thermal images for the accurate extraction of maize Tc.

## Introduction

The most important challenge for agriculture in arid and semi-arid areas worldwide is the need to produce more food under water-limited conditions (Han et al., 2018). The current food demand will double by 2050 because of projected population and socio-economic growth. For developing countries to meet this challenge, cereal yields need to increase by 40%, and net irrigation water requirements will increase by 40–50% (Atkinson et al., 2018). It is necessary to accelerate plant breeding efforts to increase potential yields and achieve maximum production per unit of applied irrigation water. Accurate measurements of crops’ responses to water stress are essential for screening drought-tolerant crop species and for achieving a delicate balance between yield and irrigation.

Currently, there are two methods for detecting water stress in crops: One is based on soil water content, and the other is based on crop parameters (Ihuoma and Madramootoo, 2017). Crop physiological changes, e.g., stomatal conductance (Gs) and leaf water potential, and biophysical changes, e.g., leaf and canopy structure, have been widely used to monitor crop water status (Gerhards et al., 2018). However, on-site measurements of soil water content and crop characteristics are time-consuming, laborious, and costly and cannot represent the spatial variability of crop water status (Campbell and Campbell, 1982; Li et al., 2010).

For decades, crop water stress has been monitored using satellite-based remote sensing images (Du et al., 2013; Calera et al., 2017; Veysi et al., 2017; Helman et al., 2018). For example, Veysi et al. (2017) evaluated the water status of a sugarcane plantation in southwest Iran using Landsat 8 thermal infrared data. The advantages of this method are that it is non-destructive and requires low labor inputs. However, satellite-based remote sensing imagery is often not suitable for monitoring crop water stress at the farm scale due to its coarse spatial resolution and homogeneity of data with large pixels (Sagan et al., 2019). In addition, cloud cover also remains a significant challenge in satellite-based remote sensing (Mulla, 2013).

In recent years, unmanned aerial vehicles (UAVs) have become an advanced field phenotyping platform to provide data with high spatio-temporal resolution. These vehicles have boosted the use of near-earth aerial imagery to monitor crop water status (Park et al., 2017; Poblete et al., 2018; Quebrajo et al., 2018; Yang et al., 2018; Zhang et al., 2019). For example, the water status of a cotton crop was evaluated using UAV thermal imagery with high spatio-temporal resolution (1-day revisits and 0.01-m resolution) in Yangling, Shaanxi, China (Bian et al., 2019). Crop water status is often monitored using UAV thermal remote sensing technology (Martínez et al., 2016; Santesteban et al., 2017; Zhang et al., 2018b; Zhang et al., 2018c), because canopy temperature (Tc) is one of the most important physiological parameters related to transpiration, leaf water potential, and Gs. When the water supply is adequate, rising environmental temperature results in increased Gs and a higher transpiration rate of crops to cool the leaves, resulting in insignificant changes in Tc (Tanner, 1963). However, under drought conditions, the leaf Gs and transpiration rate may decrease. Consequently, the Tc may increase because of the reduction in the cooling effect of transpiration (Tanner, 1963; Gates, 1968).

The use of UAV thermal remote sensing technology to monitor crop water status involves three important steps: temperature calibration, Tc extraction, and establishment of a Tc-based crop water stress indicator (Ribeiro-Gomes et al., 2017; Gerhards et al., 2018). To calibrate UAV thermal imagery, a linear regression model is often established between the measurements obtained using a handheld infrared thermometer and those obtained from UAV thermal images (Harvey et al., 2016; Bian et al., 2019; Sagan et al., 2019). For example, Yang et al. (2018) collected ground-truth temperature data for maize canopy and white-black boards to calibrate UAV thermal images. The coefficient of determination (*R*^2^) between the ground-truth temperatures and those estimated from UAV thermal images was 0.99 in the range of 25–55°C. However, due to the strong influence of environmental factors (e.g., air temperature and humidity) and the locations where images are acquired on Tc, it is important to establish specific linear regression models according to particular environmental conditions and measurement locations (Sugiura et al., 2007; Torres-Rua, 2017).

When using UAV thermal imagery to monitor crop water status before the crop reaches effective canopy cover, it is necessary to extract pure canopy pixels while avoiding the pixels of soil and other background materials in the images. There are two commonly used methods to exclude background pixels: a threshold-based approach and a co-registration approach. The threshold-based approach uses thermal imagery only, and Tc is extracted using algorithms such as Otsu and edge detection (Meron et al., 2010; Rud et al., 2014). For example, Ludovisi et al. (2017) used two alternative automatic threshold segmentation approaches (in-house algorithms in Matlab and eCognition) to extract Tc data for black poplar. Park et al. (2017) excluded ambiguously mixed pixels in the canopy-soil boundary using an edge detection method combined with Sobel and Canny algorithms in analyses of images of nectarine and peach orchards, and then they established an adaptive crop water stress index (CWSI) model. Zhang et al. (2018c) extracted Tc data for cotton using a threshold-based approach based on Otsu and canny algorithms. However, mixed pixels in thermal images can cause significant bias in Tc measurements because of the relatively low spatial resolution (from 320 × 240 to 640 × 480 pixels). To reduce bias in the extracted Tc data, a co-registration approach using both thermal and other (e.g., red–green–blue (RGB) and multispectral) imagery has been proposed. Co-registered RGB or multispectral imagery can help to mask the temperature of non-canopy features such as soil. For example, Poblete et al. (2018) proposed a method for automatic co-registration of UAV thermal and multispectral imagery to extract Tc data for a vineyard using a computer vision algorithm of modified-scale invariant feature transformation and Kmeans++ clustering. However, the wide row spacing in vineyards, e.g., 2.8 m in Baluja et al. (2012), results in small fractional canopy cover (e.g., 19% in Poblete et al., 2018). Compared with vineyards, maize crops show a wide range of fractional cover, increasing from 0 to 1 as the crop grows (Han et al., 2018). These changes and the large proportion of canopy cover may affect the extraction of maize Tc data. Therefore, the extraction of maize Tc data using a co-registration approach should be explored.

Among the Tc-based crop water stress indicators, CWSI is the most widely used. This model has been used to monitor the water status of various plants, such as maize (Irmak et al., 2000; Zia et al., 2013; Han et al., 2018; Zhang et al., 2018a), cotton (Cohen et al., 2015; Zhang et al., 2018c), grapevine (Zarco-Tejada et al., 2013; Pou et al., 2014; Bellvert et al., 2015), peach (Wang and Gartung, 2010; Paltineanu et al., 2013; Bellvert et al., 2014), and olive (Berni et al., 2009; Agam et al., 2013a; Agam et al., 2013b). There are two widely used CWSI models: the empirical model proposed by Idso et al. (1981) and the theoretical model proposed by Jackson et al. (1981). Although the empirical model has the advantage of being easier to establish after the determination of non-water-stress and non-transpiring (stomata fully closed) baselines, it still requires at least three parameters, i.e., Tc, air temperature, and relative humidity, or wet and dry reference positions (Zhang et al., 2019). Some researchers have attempted to reduce the number of parameters required to calculate crop water stress indicators. For example, Taghvaeian et al. (2014) compared the performances of CWSI and degrees above non-stress (DANS) models to estimate the water stress of sunflower crops in northern Colorado. Their results showed that DANS based solely on Tc and extracted by a simple subtraction could be used to monitor water stress and schedule irrigation for water-deficient sunflower crops in arid and semi-arid areas. Han et al. (2016) developed a new crop water stress indicator, standard deviation of Tc (CTSD), within a thermal image to monitor the water stress of maize crops in Greeley, Colorado, USA. Soil water deficit, leaf water potential, Gs, and other crop water stress indicators were shown to be highly correlated with CTSD. Their results suggested that the CTSD model has good potential for scheduling irrigation because it relies only on Tc and is easy to calculate. Zhang et al. (2018b) proposed that Tc characteristics obtained from thermal images, including CTSD and coefficient of variation of Tc (CTCV), could be used to monitor water stress of cotton crops in Yangling, Shaanxi, China.

The aim of this study was to explore the use of the co-registration approach to extract maize Tc data and monitor maize water stress at the farm scale. We obtained and analyzed UAV-based thermal and RGB images, and we evaluated the performance of Tc and Tc-based indicators (CTSD, DANS, and CTCV) for monitoring maize water stress.

## Materials and Methods

### Study Site

**Figure 1:**
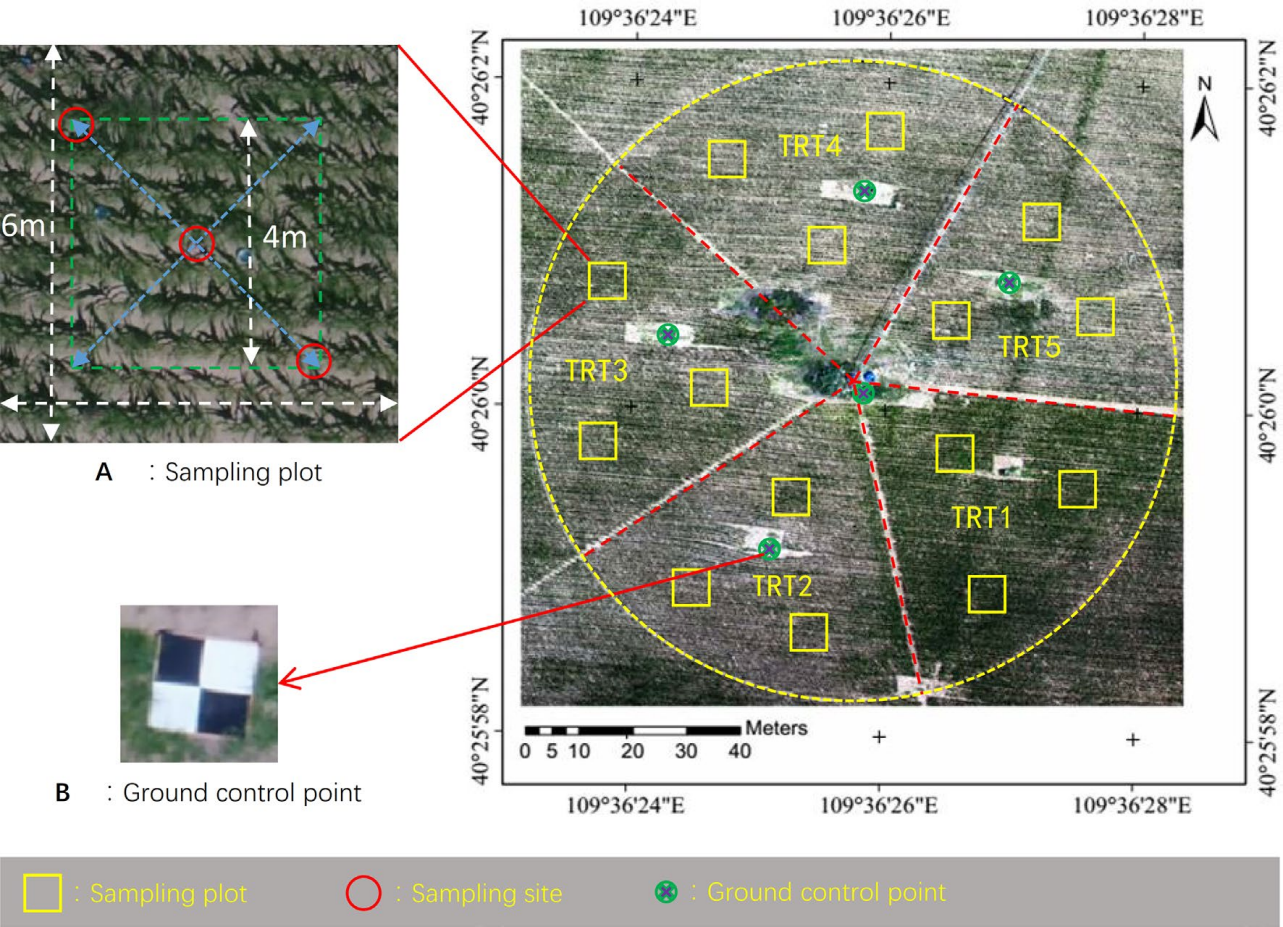
Aerial view of the experimental field. Shown are treatment region division, location of sampling plots, sampling sites **(A)**, and ground control sites **(B)**.

This study was conducted in a 1.13-ha research field (40°26′0.29″N, 109°36′25.99″E, elev. 1,010 m), located in Ordos, Inner Mongolia, the North China Plain, where rainfall cannot meet crop water requirements. The study field was divided into five regions with five different irrigation treatments (TRTs). Three areas measuring 6 × 6 m^2^ within each region were selected as sampling plots. In each plot, three sampling sites were selected for data collection (yellow rectangles in **Figure 1**). At the effective rooting depth (0–90 cm), the volumetric soil water content at field capacity was 13%, and permanent wilting point was 5.6%. More detailed information on the soil at the site is provided in Zhang et al. (2019). Maize (*Zea mays* cv. Junkai 918) was planted on May 11, 2018 [day of year (DOY) 131], with a row spacing of 0.58 m, plant spacing of 0.25 m, and east–west row direction. The maize plants emerged on May 18, headed on July 21, and were harvested on September 10, 2018 (silage) after a 115-day life span.

### Experimental Design

During the late vegetative stage (V7–VT, DOY 184–206) before full canopy cover, three different levels of irrigation were applied in TRTs 1–5; full irrigation (FI, TRT 1), moderate deficit irrigation (MDI, TRTs 2 and 4), and severe deficit irrigation (SDI, TRTs 3 and 5). Non-stressed crop evapotranspiration was estimated by the reference evapotranspiration and single crop coefficient approach (Allen et al., 1998). The crop coefficient was 0.66, 1.31, and 0.54 in the initial, mid-season, and late-season developmental stages (alfalfa-based), respectively. The maize plants were irrigated using a center pivot sprinkler system (Valmont Industries, Inc., Omaha, USA) with two spans and one end gun (total length, 143.7 m). Detailed information about the center pivot sprinkler system is provided elsewhere (Li et al., 2018). The coefficient of uniformity for the first span (research field) using R3000 sprinklers was 82.7% or 88.3% at 20% or 40% of full walking speed, respectively, as calculated using the modified formula of Heermann and Hein (1968). The amount of water applied to each TRT was measured and recorded using a MIK-2000H flow meter (Meacon Automation Technology Co., Ltd, Hangzhou, China). To eliminate interference from nutritional stress and weeds, fertilizer and herbicide were applied according to the local cultivation practices.

**Figure 2:**
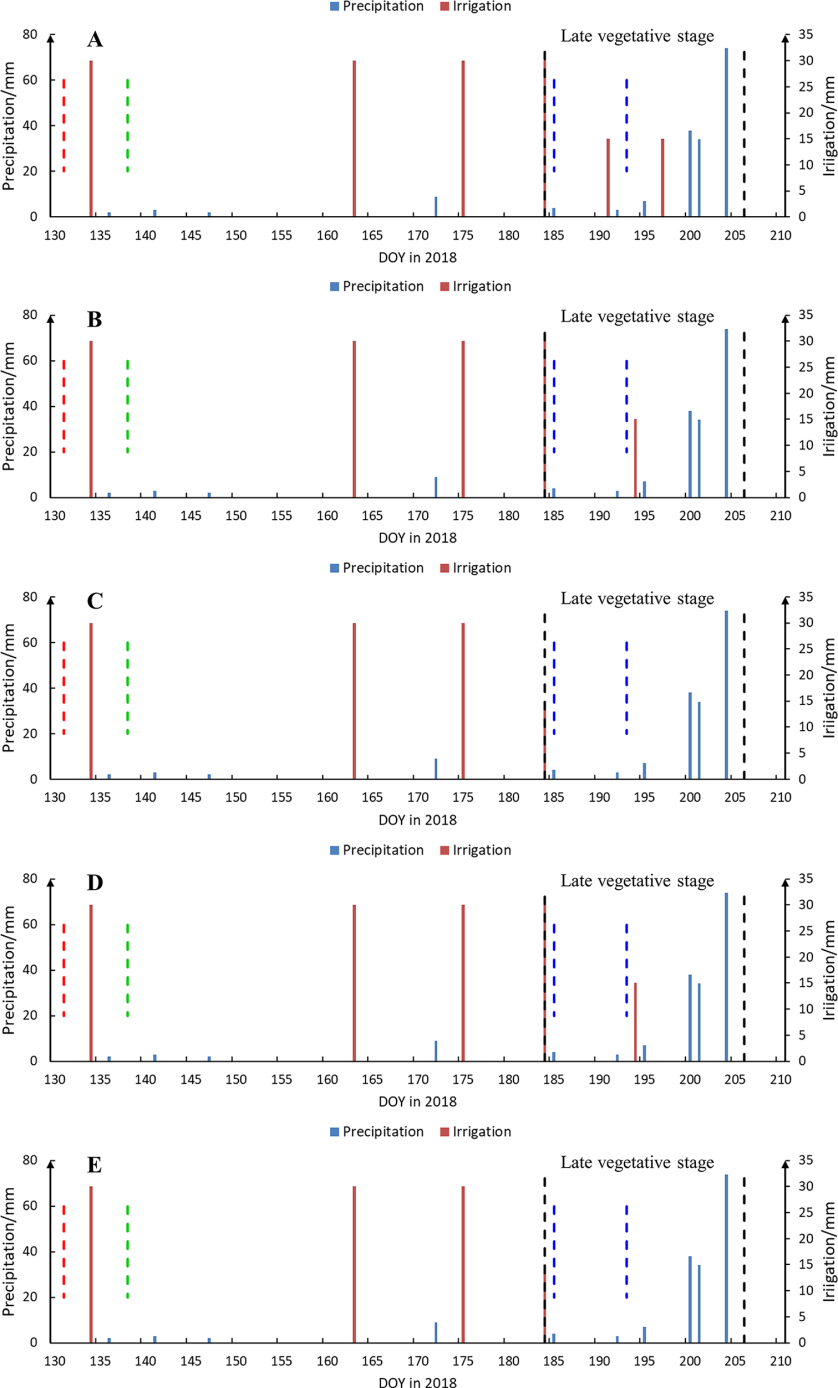
Dates (day of year (DOY)) and amounts of precipitation (blue solid line) and irrigation (red solid line) events from seeding to tassel in 2018. Panels of **(A**–**E)** are for treatments 1–5, respectively. The dotted lines indicate dates of seeding (red) and emergence (green), the boundaries of late vegetative stage (black), and the date of data collection (blue). DOY 185 was rainy before dawn and was sunny during the daytime.

**Figure 2** shows the specific dates and amounts of precipitation and irrigation events. Before the start of deficit irrigation (DOY 184), all treatments (TRTs 1–5) received an equal amount of irrigation provided in three applications (total amount, 90 mm). The purpose of this early season irrigation was to provide sufficient water in all treatments so that plants would emerge and grow as uniformly as possible before imposing water deficit at different levels. During DOY 131–184, the total amount of precipitation was 16 mm. During DOY 184–197, TRT 1, TRTs 2 and 4, and TRTs 3 and 5 received 60, 45, and 15 mm of water, applied in three, two, and one applications, respectively. During this time, there were three rain events (in total, 14-mm precipitation). During DOY 198–206, there were three unusually heavy rain events (in total, 146-mm precipitation).

### Field and Meteorological Data Collection

**Figure 3:**
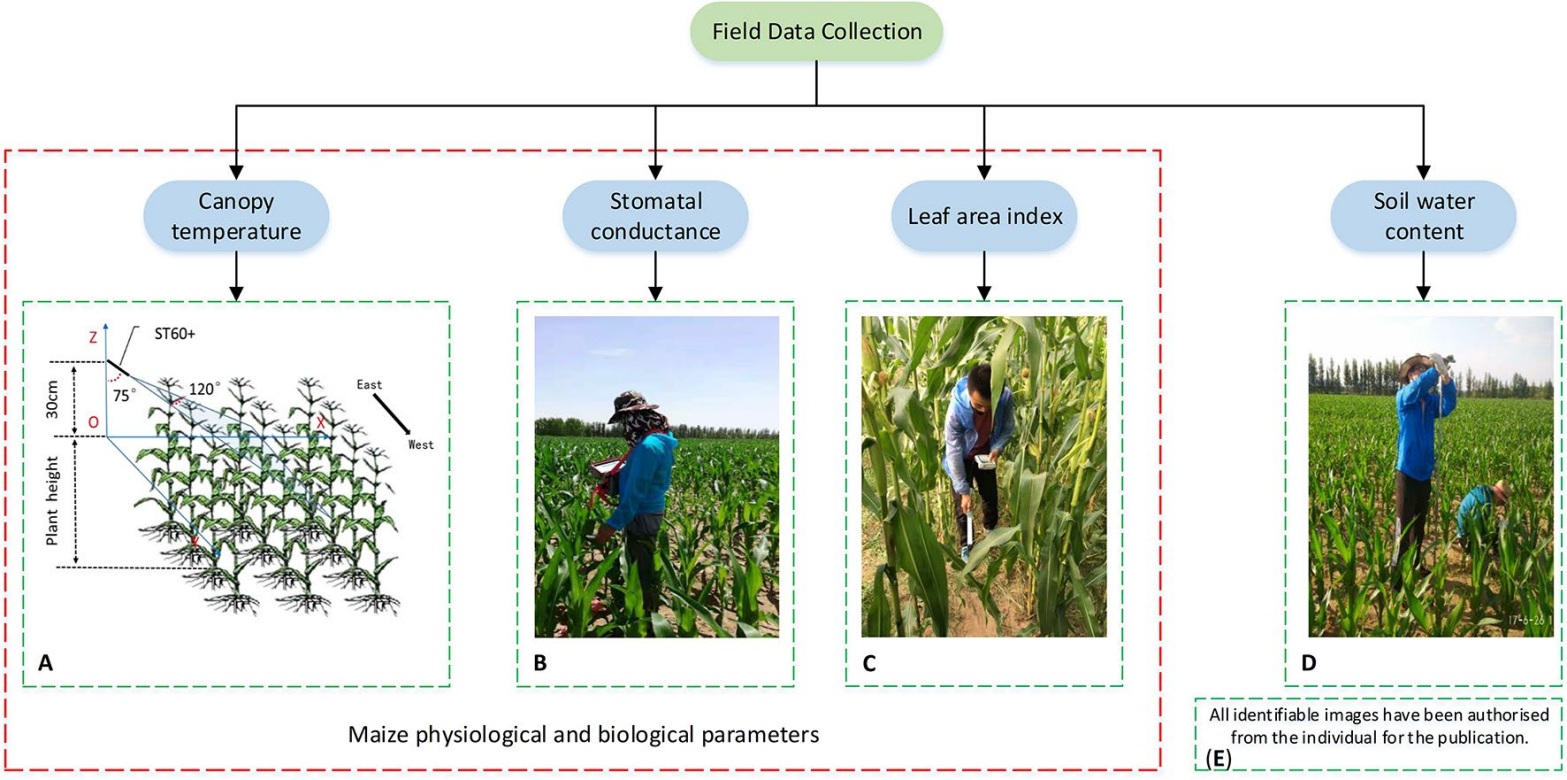
Schematic indicating the field data collection within each sampling site, including measurements of canopy temperature **(A)**, stomatal conductance **(B)**, leaf area index **(C)**, soil water content **(D)**, and written informed consent of identifiable imagery **(E)**.

**Figure 3** shows the field data collected including ground-truth Tc (**Figure 3A**), stomatal conductance (Gs, **Figure 3B**), leaf area index (LAI, **Figure 3C**), and soil water content (SWC, **Figure 3D**). On DOY 185 and 193 (sunny days), ground-truth Tc, Gs, and UAV images were collected between 11:00 and 13:00 (Chinese standard time). The LAI was measured at 2 h before sunset, to avoid the influence of direct sunlight. The SWC data were collected in the afternoon. At each sampling plot, ground-truth Tc, Gs, and LAI measurements were taken at three sampling sites, and the average values of these three readings were used to represent the sampling plot. A total of 45 data sets of samples (ground-truth Tc, Gs, and LAI) were obtained on each sampling day.

Ground-truth Tc was measured by a handheld infrared thermometer (RAYTEK, ST60+, Raytek Inc., Santa Cruz, USA) with a temperature range of 32–600°C and a spectral range of 8–14 µm. The measurement error is ±1% of the reading or ±1°C, whichever value is larger. The emissivity value was set to 0.97. To avoid interference from the soil, the infrared thermometer was moved across the canopy (at about 120°) perpendicular to the row at 30 cm above the canopy with a horizontal angle of 15° (**Figure 3A**). The Gs was measured using an AP4 porometer (Delta-T Devices, Burwell, Cambridge, CB25 0EJ, UK) with a measurement range of 5.0–1,200 mmol·m^−2^·s^−1^ and a measurement accuracy of ±10% (5–800 mmol·m^−2^·s^−1^) or ±20% (800–1200 mmol·m^−2^·s^−1^). At each sampling site, measurements were conducted on the upper side of two fully collared sunlit leaves. The LAI was measured using an LAI-2200C plant canopy analyzer (LI-COR, USA). At each sampling site, radiation values were measured at the top of canopy and at four marked points under the canopy.

The SWC was measured at the center of each sampling plot using the traditional gravimetric method. The SWC was determined at six depths (10, 20, 30, 45, 60, and 90 cm) in each plot. Details of SWC measurements are provided in Zhang et al. (2019). The meteorological data were measured by an automated weather station located in a 1-ha alfalfa field adjacent to the research field. The meteorological data included rainfall, air temperature, relative humidity, net solar radiation, and wind speed (at 2 m above the ground). Except for rainfall, other meteorological factors were measured at 30-min intervals. The mean daily air temperature, relative humidity, net solar radiation, and wind speed during the maize late vegetative stage were 22°C, 73%, 101 W/m^2^, and 0.7 m/s, respectively.

### UAV System and Data Collection

#### UAV Thermal and RGB Imaging Systems

**Figure 4:**
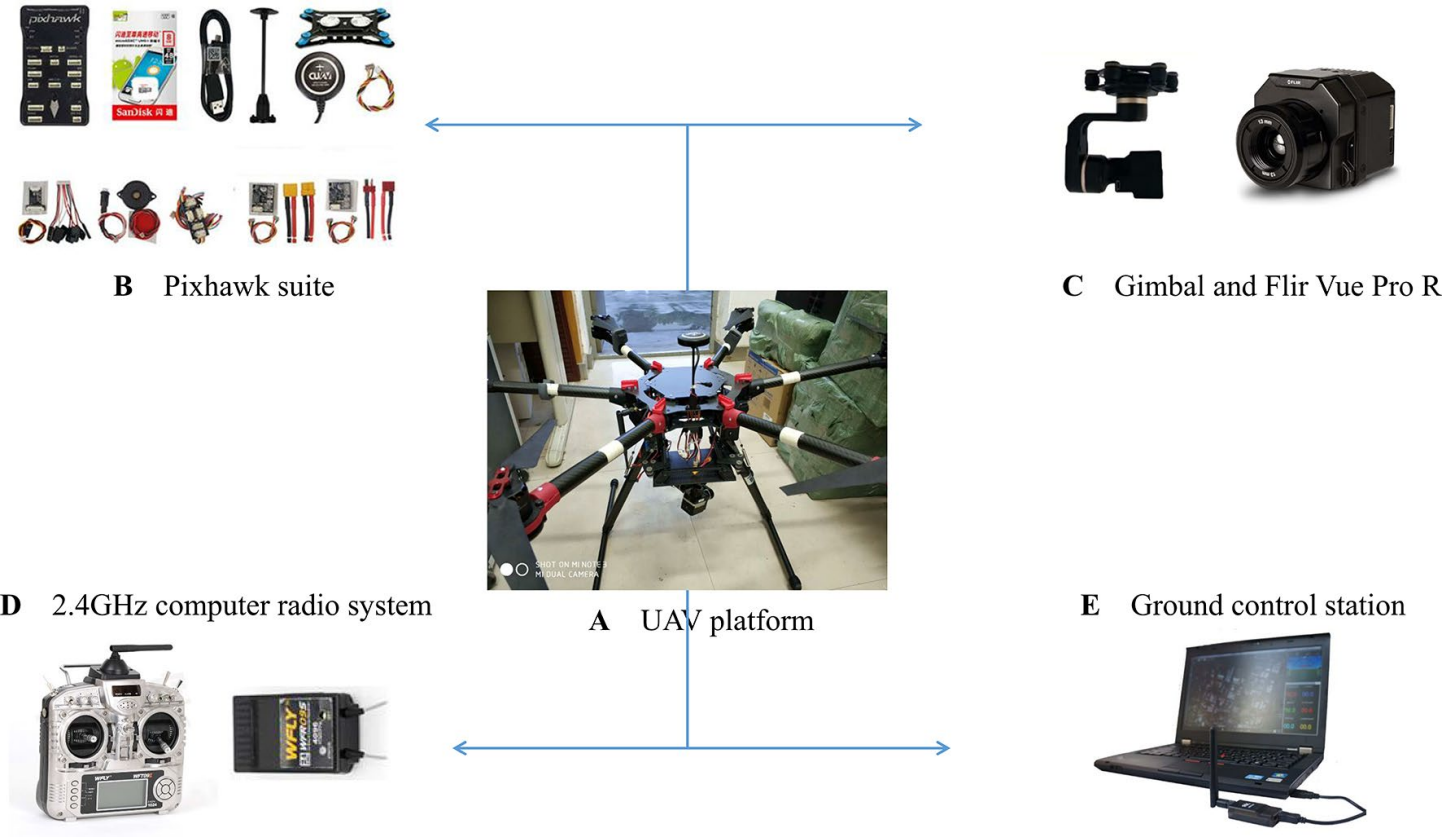
Schematic indicating the main components of unmanned aerial vehicle (UAV) thermal remote sensing system developed in this study, including UAV platform **(A)**, pixhawk suite **(B)**, gimbal and Flir Vue Pro R camera **(C)**, 2.4GHz computer radio system **(D)**, and ground control station **(E)**.

In this study, a hexa-copter UAV thermal remote sensing system (**Figure 4**) was developed with a PIXHAWK autopilot (CUAV, Guangzhou, China), a FLIR Vue Pro R 640 thermal camera (FLIR Systems, Wilsonville, OR, USA), and a Feiyu brushless gimbal (Moyouzhijia, Huizhou, China). The main technical parameters are shown in **Table 1**. The FLIR Vue Pro R 640 is a small radiometric thermal sensor designed for UAV integration and data collection. It has a claimed accuracy of ±5°C and thermal sensitivity of 0.05°C. It is easy to operate with many MAVLink autopilots (e.g., PIXHAWK) using the included accessory cable and can be triggered based on time intervals or from waypoints within the UAV flight plan. Information of GPS locations for each image was obtained from PIXHAWK during collection. The configuration was set using the FLIR UAS mobile application that connects to the camera *via* Bluetooth. Flight planning was conducted with ground control station software, Mission Planner, which allows the user to generate a route of waypoints as a function of the field of view of the sensor, degree of overlap between images, and ground resolution. Mission Planner also displays real-time flight data.

**Table 1:** Main parameters of UAV thermal and RGB image acquisition system.

Parameter		Value
UAV thermal image acquisition system	Wheelbase	900 mm
	Takeoff weight	6 kg
	Payload	2 kg
	Flight time	18 min
	Communication radius	3 km
	Speed	5 m/s
	Imager resolution	640 × 512 pixels
	Data format	14-bit Tiff
	Spectral bands	7.5–13.5 µm
	Frame rate	9 Hz
	Lens focal length	13 mm
	Lens field of view	45° × 37°
	Accuracy	(±) 5°C
	Thermal sensitivity (NETD)	0.05°C
	Weight	< 115 g
	Dimension	63 mm × 44.4 mm × 44.4 mm
UAV RGB image acquisition system	Wheelbase	350 mm
	Weight	1,388 g
	Flight time	30 min
	Communication radius	5 km
	Speed	<72 km/s
	Imager resolution	4,864 × 3,648 pixels
	Lens focal length	8.8 mm/24 mm
	Lens field of view	84°
	Image sensor	1-in. CMOS
	RGB color space	sRGB
	ISO range	100–12,800
	Shutter speed	8–1/8,000 s
	Image format	JPEG; DNG

UAV, unmanned aerial vehicle; RGB, red–green–blue; CMOS, complementary metal-oxide semiconductor.

A quad-rotor UAV RGB remote sensing system, DJI Phantom 4 Pro (Shenzhen Dajiang Baiwang Technology Co., Ltd, China) was used to collect RGB images. This UAV system has an integrated camera with a 1-in. complementary metal-oxide semiconductor sensor that captures RGB spectral information. The camera has an 84° field of view lens with an f/2.8 aperture and a resolution of 4,864 × 3,648 pixels. This lens has been especially designed to eliminate image distortion. **Table 1** gives detailed information about the digital camera and UAV system.

#### Acquisition and Pretreatment of UAV Thermal and RGB Images

**Figure 5:**
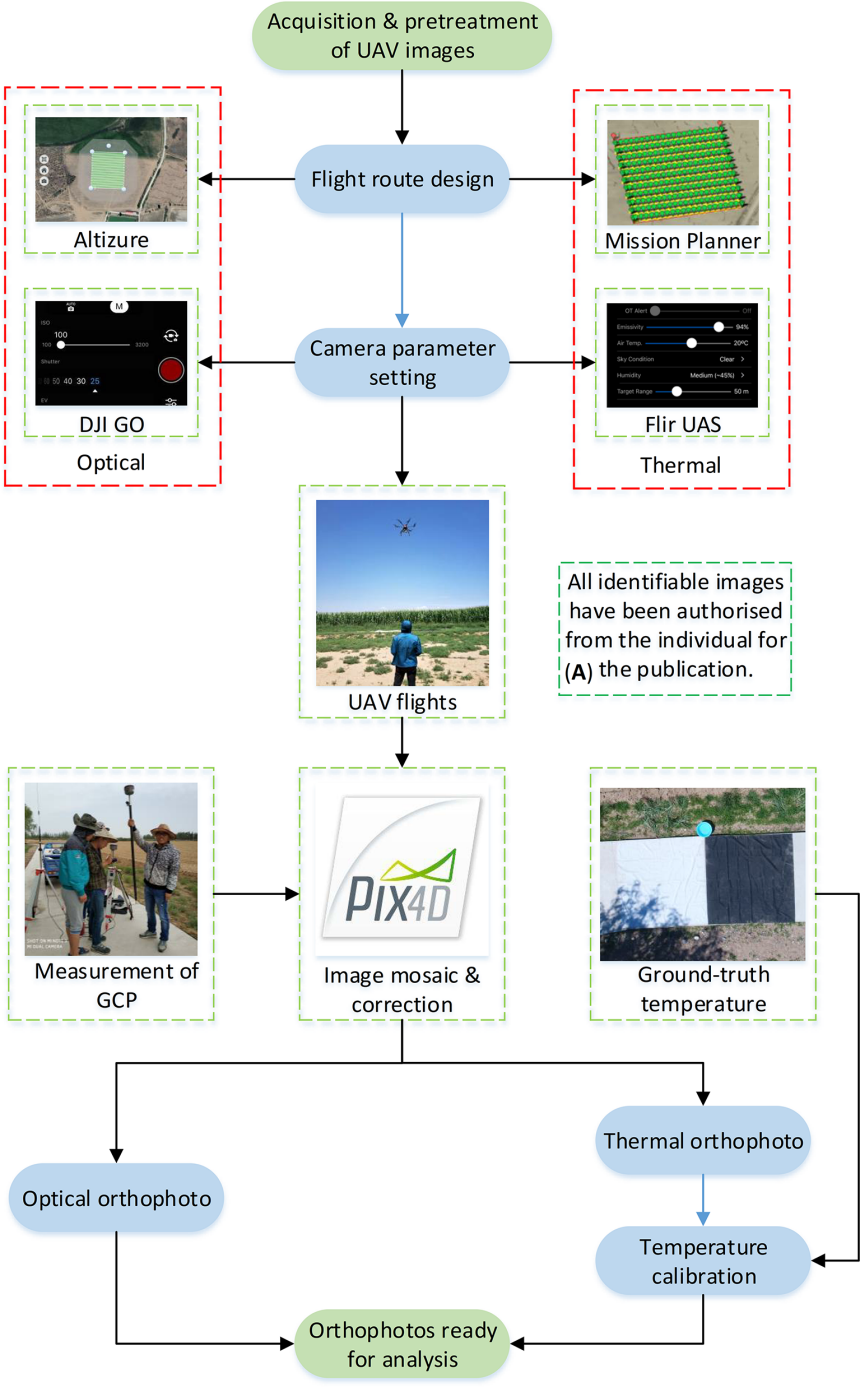
The acquisition and pretreatment of unmanned aerial vehicle (UAV) thermal and red–green–blue (RGB) imagery, including flight route design, camera parameter setting, UAV fights, image mosaicking and correction, and temperature calibration (thermal). **(A)** Written informed consent of identifiable imagery.

**Figure 5** shows the main procedures for acquisition and pretreatment of UAV thermal and RGB images. On DOY 185 and 193 (sunny days) between 11:00 and 13:00, thermal images were obtained with the FLIR Vue Pro R 640 camera lens facing downward vertically, and with 85% front and side overlap. The flight height, speed, and ground sample distance were 60 m (relative flying height), 5 m/s, and 7.8 cm, respectively. Before collecting thermal images of the maize canopy, images of black (reflectivity 3%) and white (reflectivity 58%) diffuse boards (size 3 m × 3 m, Group VIII, USA) and water were captured at relative flying heights of 10, 20, 30, 40, 50, and 60 m. At the same time, the temperatures of the above three objects were measured using a handheld infrared thermometer (RAYTEK ST60+) for temperature calibration. Before the flight, the emissivity and image format were set to 0.97 and 14-bit Tiff. The outdoor mode was selected; and parameters of weather conditions, atmosphere temperature, and humidity were set to actual conditions. The infrared thermal camera was pre-heated for about 10 min to reduce systematic error (Yang et al., 2018).

On DOY 185 and 193 (sunny days) between 11:00 and 13:00, RGB images of maize were acquired using the digital camera mounted on a DJI Phantom 4 Pro. Flights were controlled by Altizure software (Everest Innovation Technology Ltd, Hong Kong, China), which directed the UAV to fly along a serpentine image acquisition plan at height of 50 m and a speed of 2.5 m/s with the camera facing downwards. The overlap of front and side images was 90%. The ground sample distance was 1.25 cm. The parameters of ISO, white balance, and shutter were set to 400, 1/1,250, and sunny, respectively.

After images were acquired, mosaic processing was performed using Pix4DMapper software (Pix4DInc., Lausanne, Switzerland), which is specifically designed to process UAV images using techniques rooted in both computer vision and photogrammetry (Turner et al., 2012). Thermal and RGB orthomasaics were geo-referenced using five ground control points (**Figure 1**) whose coordinates were measured using a KOLIDA RTK differential GNSS device (KOLIDA Instrument Co., Ltd, Guangzhou, China).

### Tc Extraction Method

**Figure 6:**
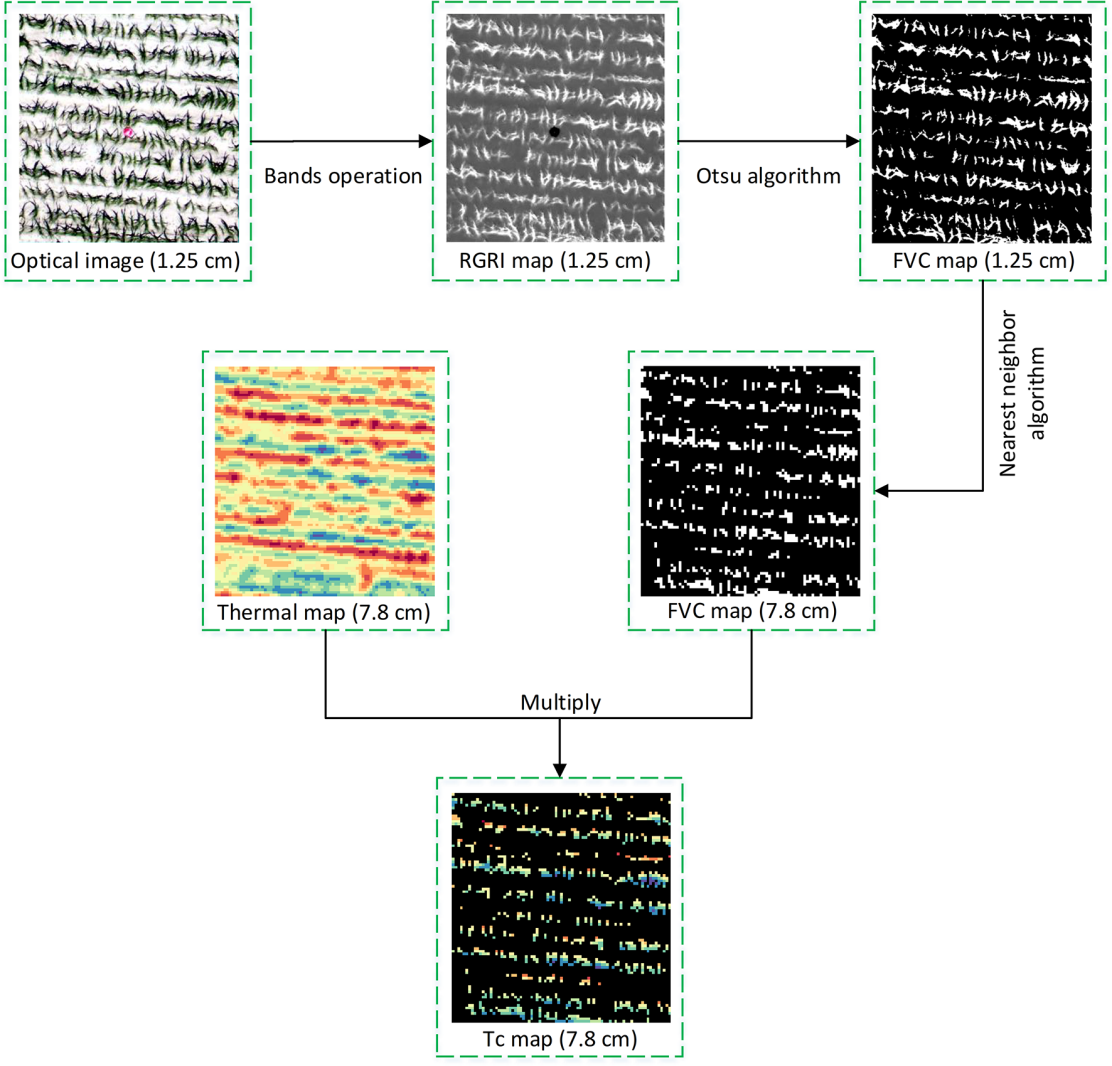
The main steps of the co-registration approach (red–green ratio index (RGRI)-Otsu method proposed in this study) using both unmanned aerial vehicle (UAV) thermal and red–green–blue (RGB) remote sensing imagery. FVC (fractional vegetation cover) and RGRI (red–green ratio index) were derived from optical image.

We used a co-registration approach (the red–green ratio index (RGRI)-Otsu method proposed in this study) to analyze UAV thermal and RGB images to extract maize Tc at the late vegetative stage (**Figure 6**). This approach involved two key steps: First, we extracted the maize fractional vegetation cover (FVC) on the basis of UAV RGB images; and second, we resampled the spatial resolution of the FVC map to match the scale of the thermal images. To obtain the FVC map, the Otsu algorithm was applied to the RGRI (Verrelst et al., 2008) map. The RGRI was calculated using equation (1):

(1) $$RGRI=\frac{R}{G},$$

where *R* and *G* represent the digital numbers of red and green bands. During the extraction of the FVC map, soil or other background pixels were flagged as 0 and maize pixels as 1. The result was an FVC map with a spatial resolution of 1.25 cm. During downscaling, the spatial resolution of the FVC map was resampled from 1.25 to 7.8 cm by using the nearest-neighbor interpolation algorithm. The nearest-neighbor interpolation is the simplest and fastest implementation and sets the pixel value of each point of the target image to the nearest point in the source image, without producing mixed pixels. This decreases the effect of mixed pixels on Tc extraction. Finally, multiplication between the FVC map (7.8 cm) and thermal image (7.8 cm) was adopted to extract a Tc map. The entire process of the RGRI-Otsu method was implemented by programming in R language (R-3.4.3, https://www.r-project.org/).

### Tc-Based Crop Water Stress Indicators

To reduce the number of parameters required for crop water stress monitoring, we chose four indices i.e., Tc, DANS, CTSD, and CTCV, which only need Tc. The values of DANS, CTSD, and CTCV were calculated using equations (2)–(4):

(2) $$DANS=\{\begin{array}{ll} \bar{Tc}-T_{NS}\; & \bar{,Tc}>28^{\circ }C\\ 0 & \bar{,T_{C}}\leq 28^{\circ }C \end{array}$$

(3) $$CTSD=\sqrt{\left[{\left(Tc_{1}-\bar{Tc}\right)}^{2}+{\left(Tc_{2}-\bar{Tc}\right)}^{2}+...+{\left(Tc_{n}-\bar{Tc}\right)}^{2}\right]/n}$$

(4) $$CTCV=\frac{CTSD}{\bar{Tc}}$$

where $\bar{Tc}$ is the mean maize Tc within a sampling plot derived by the RGRI-Otsu method, *T_NS_* is the non-stressed maize Tc, *Tc_i_* (*i* = 1, 2, …, *n*) is the actual Tc of each maize canopy pixel within a sampling plot, and *n* is number of maize canopy pixels within a sampling plot. Estimation of DANS requires appropriate selection of *T_NS_*. For maize, 28°C has been suggested by the ARS Plant Stress and Water Conservation Laboratory (Evett et al., 2000; DeJonge et al., 2015).

### Statistical Analysis

For statistical analysis, ground-truth Tc values were compared with the extraction results of maize Tc, and measured Gs values were used to evaluate the performance of the four Tc-based crop water stress indicators. Specifically, linear regression models were used with the coefficient of determination (*R*^2^) and the root mean square error (RMSE) calculated for comparisons. The regressions were implemented by using R programming language and the lm() function.

## Results

### Distribution of Ground-Truth Tc, Gs, and LAI

**Figure 7:**
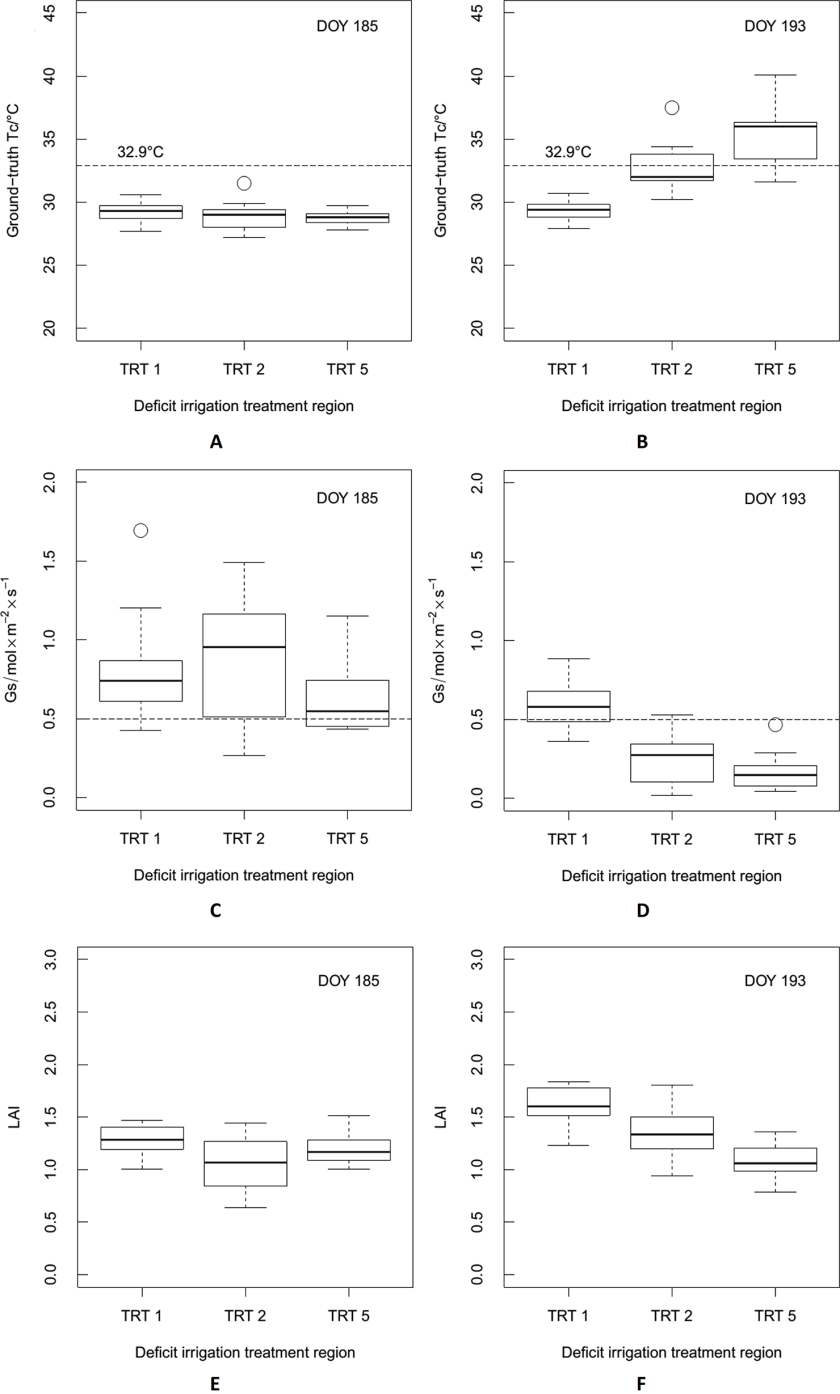
The distributions of ground-truth canopy temperature (Tc), stomatal conductance (Gs), and leaf area index (LAI) for deficit irrigation treatments at three different levels (TRT 1, TRT 2, and TRT 5), based on data acquired on day of year (DOY) 185 and DOY 193, respectively. TRT 1, TRT 2, and TRT 5 were full irrigation (FI), moderate deficit irrigation (MDI), and severe deficit irrigation (SDI), respectively. Panels **(A)**, **(B)**, and **(C)** were ground-truth Tc, Gs, and LAI acquired on DOY 185, respectively, while panels **(D)**, **(E)**, and **(F)** were ground-truth Tc, Gs, and LAI acquired on DOY 193, respectively.

**Figure 7** shows the distributions of ground-truth Tc, Gs, and LAI, for TRTs 1, 2, and 5 based on data acquired on DOY 185 and 193, respectively. As shown in **Figure 7A, E**, because of the irrigation event on DOY 184 when the deficit irrigation treatments began (**Figure 2**), there was no obvious difference in the distributions of ground-truth Tc and LAI among the three different irrigation treatments. The average ground-truth Tc values were 29.2°C, 28.9°C, and 28.7°C, and LAI were 1.3, 1.1, and 1.2 for TRTs 1 (FI), 2 (MDI), and 5 (SDI), respectively. The Gs values for TRTs 1, 2, and 5 were higher than 0.50 mol·m^−2^·s^−1^, which is the non-stress baseline for maize (Han et al., 2016) (**Figure 7C**, DOY 185). However, with prolonged water deficit, clear gradients in the distributions of ground-truth Tc, Gs, and LAI were detected among the irrigation treatments (**Figures 7B, D, F**, DOY 193). As shown in **Figure 7B**, the ground-truth Tc of TRT 1 was below 30.0°C. The Tc increased with increasing severity of drought stress. The average ground-truth Tc in TRTs 1, 2, and 5 was 29.4°C, 32.7°C, and 35.3°C, respectively. As shown in **Figure 7D**, the Gs of TRT 1 was higher than 0.50 mol·m^−2^·s^−1^, indicating no water stress. The Gs tended to decrease with increasing severity of drought stress. The average Gs for TRTs 1, 2, and 5 was 0.60, 0.24, and 0.17 mol·m^−2^·s^−1^, respectively. As shown in **Figure 7F**, the highest LAI was in TRT 1, and LAI tended to decrease with increasing severity of drought stress (average LAI of 1.6, 1.4, and 1.1 in TRTs 1, 2, and 5, respectively).

### Calibration of Temperature Derived From UAV Thermal Images

**Figure 8:**
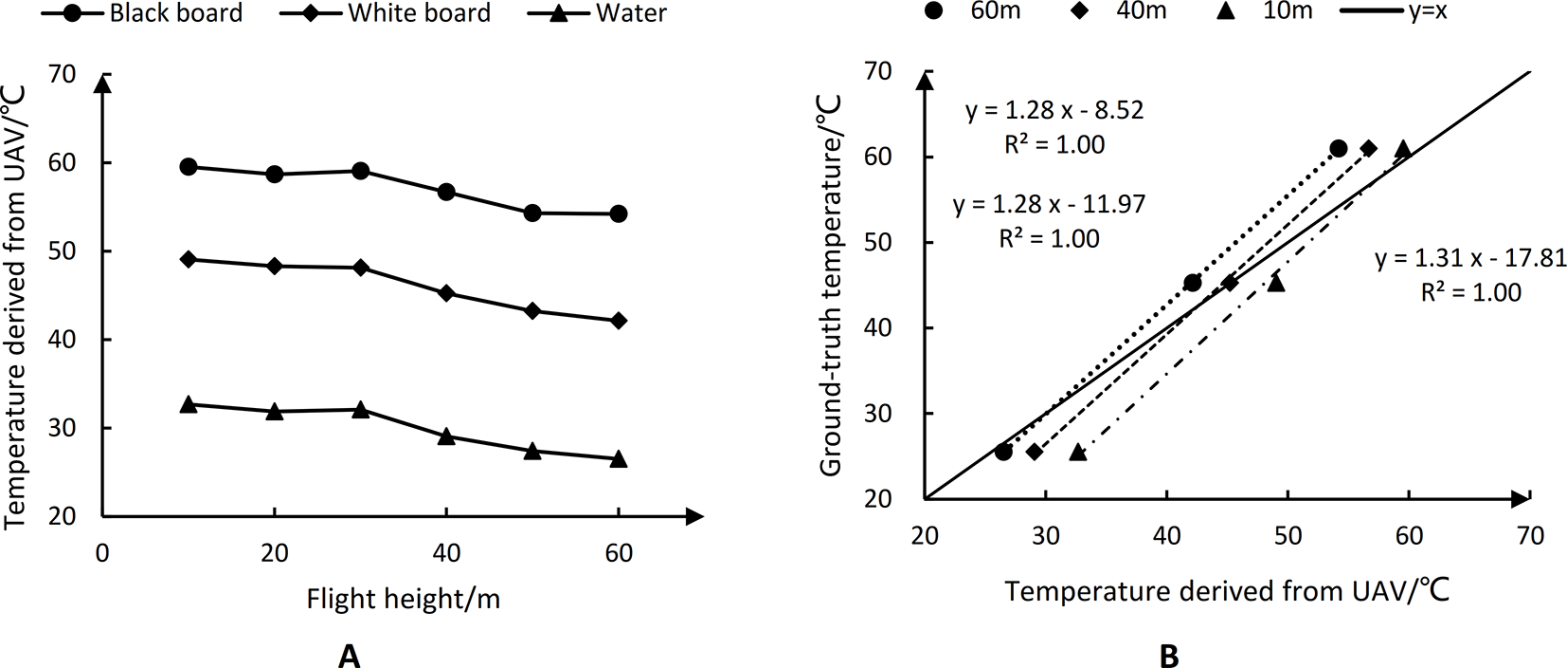
The temperature variations of water and black-white diffuse boards derived from unmanned aerial vehicle (UAV) thermal imagery at different flight heights **(A)**; regression model between temperatures derived from UAV thermal imagery and ground-truth temperatures **(B)**.

**Figure 8A** shows the temperature variations of water and diffuse boards derived from UAV thermal images captured at different flight heights (10, 20, 30, 40, 50, and 60 m) on DOY 193. With increasing height of image acquisition (10–60 m), the temperatures of the three objects on the ground showed a clear downward trend. The difference between the highest and lowest estimated temperatures for water and the two diffuse boards were 5.35°C, 6.91°C, and 6.17°C, respectively, indicating that the acquisition height of UAV thermal images affects the accuracy of the derived temperatures. As the height increased, the temperatures derived from UAV thermal images significantly decreased. **Figure 8B** shows the relationships between temperatures derived from the UAV thermal images and ground-truth measurements. At different flight heights (10, 40, and 60 m), there were good linear correlations with a slope about 1.30. However, as the height increased, the intercept of the linear correlation changed markedly and became smaller. This provided more evidence that the temperature obtained by the thermal infrared camera significantly decreased as the flight height increased.

### Extraction of Maize Tc

**Figure 9:**
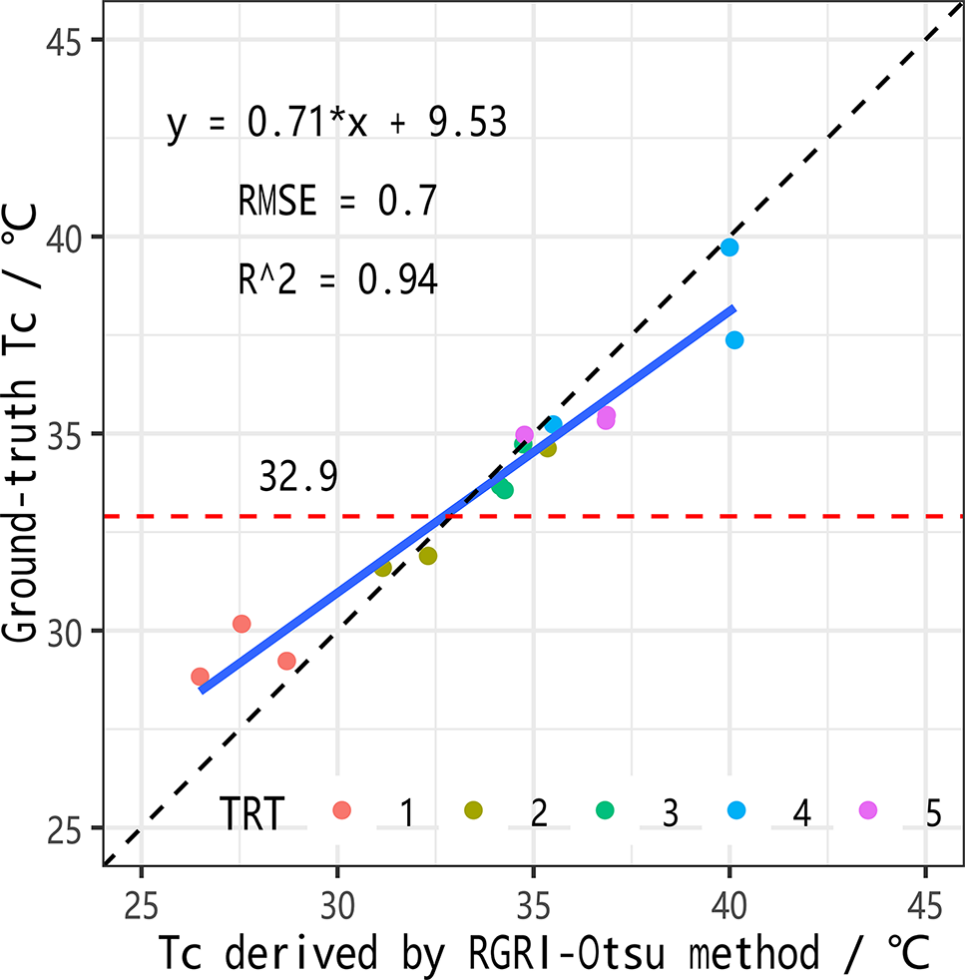
Linear regression model between maize canopy temperatures (Tc) extracted by the co-registration approach (red–green ratio index (RGRI)-Otsu method) and by ground-truth.

As shown in **Figure 7C**, there was no water stress in the three irrigation treatments on DOY 185, with Gs values above the non-stress baseline for maize (0.50 mol·m^−2^·s^−1^). Therefore, to better describe the relationship between ground-truth Tc and Tc derived from UAV thermal images, data acquired on DOY 193 for maize under different levels of water stress in three irrigation treatments were used in a linear regression analysis (**Figure 9**). There was a high correlation with an *R*^2^ value of 0.94 (*n* = 15) and an RMSE value of 0.7°C. At the same time, there was a significant deviation from the 1:1 line with slope and intercept of 0.71 and 9.53, respectively. Specifically, when the ground-truth Tc was less than 32.9°C, a lower Tc was obtained by RGRI-Otsu extraction, and when the ground-truth Tc was greater than 32.9°C, a higher Tc was obtained by RGRI-Otsu extraction. Therefore, to obtain more accurate values, the RGRI-Otsu extracted Tc should be modified accordingly based on the linear regression model. Similar deviations have been found in other studies (Baluja et al., 2012; Yang et al., 2018; Sagan et al., 2019).

### Relationships Between Tc-Based Crop Water Stress Indicators and Gs and LAI of Maize

**Figure 10:**
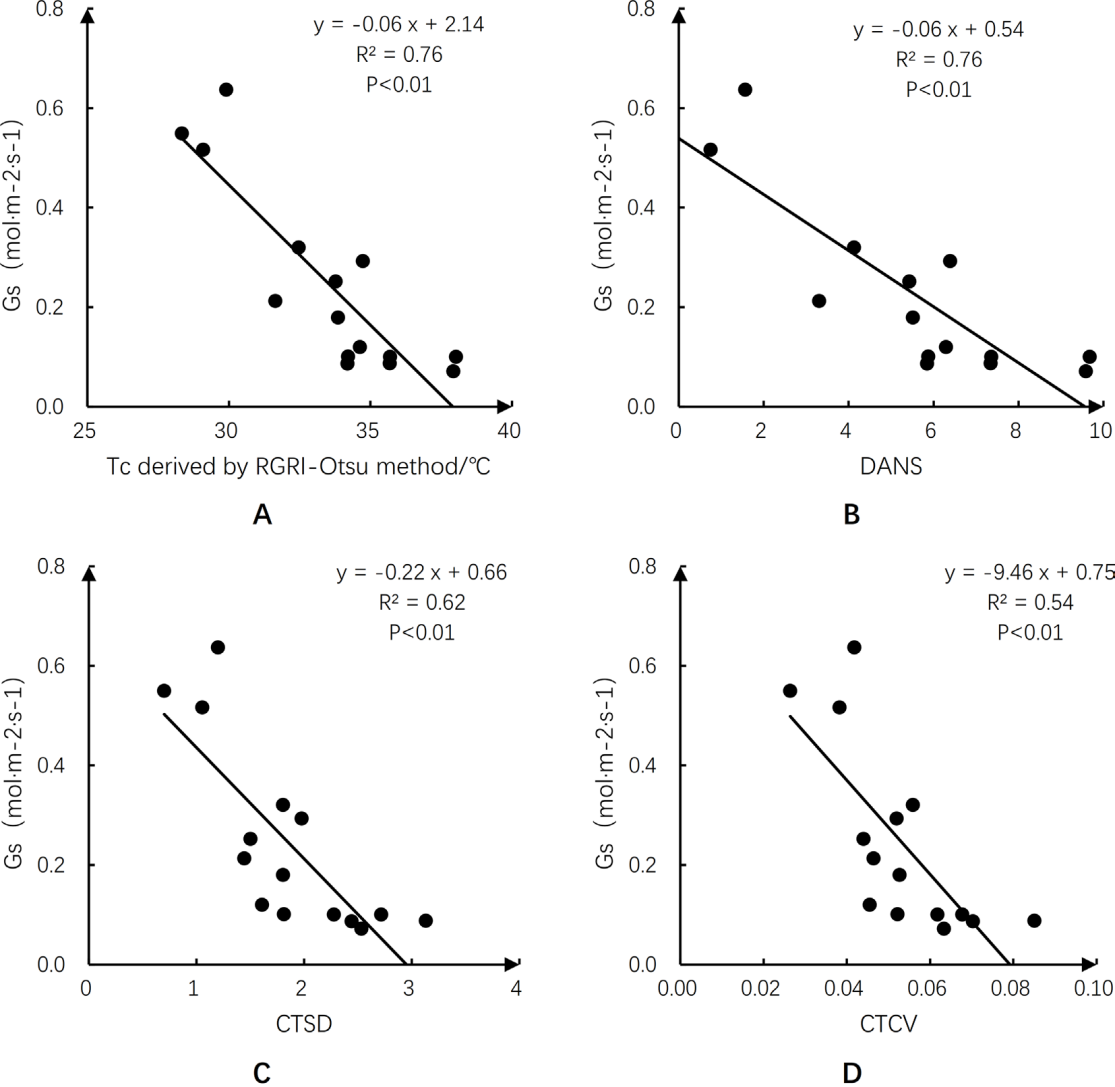
Relationships of canopy temperature (Tc)-based crop water stress indicators with stomatal conductance (Gs). The data were acquired on day of year (DOY) 193. Panel **(A)** for canopy temperature (Tc) derived by red–green ratio index (RGRI)-Otsu method; **(B)** for degrees above non-stress (DANS); **(C)** for standard deviation of canopy temperature (CTSD); and **(D)** for canopy temperature coefficient of variation (CTCV).

**Figure 11:**
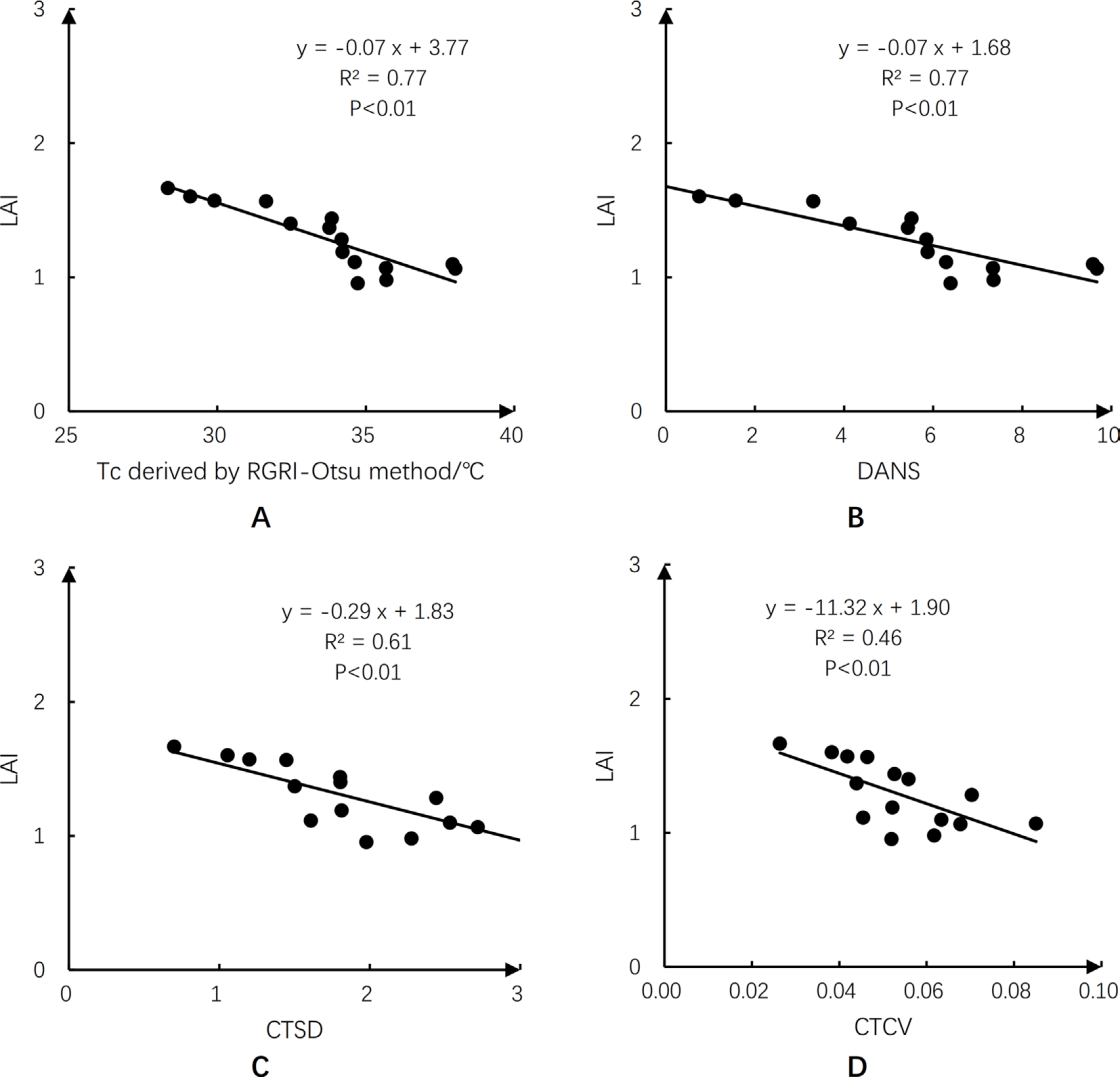
Same as **Figure 10**, but for leaf area index (LAI).

**Figure 10** illustrates the relationships between Tc-based crop water stress indicators and Gs. There were significant negative correlations between Gs and Tc, DANS, CTSD, and CTCV (*p* < 0.01), with the largest *R*^2^ of 0.76 for Tc and DANS, and lower *R*^2^ of 0.62 and 0.54 for CTSD and CTCV, respectively. Plant leaves curl under water stress, resulting in lower LAI (Taghvaeian et al., 2014). Therefore, the relationships between Tc-based crop water stress indicators and LAI were analyzed (**Figure 11**). There were significant negative correlations between LAI and Tc, DANS, CTSD, and CTCV (*p* < 0.01). When there was no or mild water stress and high LAI values (no leaf rolling), the four Tc-based water stress indicators had smaller values. When there was water stress and LAI values were lower (caused by leaf rolling), the four Tc-based water stress indicators had larger values; the *R*^2^ values were 0.77 for Tc and DANS, and 0.61 and 0.46 for CTSD and CTCV, respectively.

### Relationships Between Tc-Based Crop Water Stress Indicators and SWC

In general, Tc, DANS, CTSD, and CTCV showed significant (*p* < 0.05) correlations with SWC at the depths of 10, 20, and 30 cm, except for CTSD and CTCV with SWC at 10 cm (**Table 2**). In contrast, there were no significant correlations between the four indicators and SWC at the soil depths of 45, 60, and 90 cm. Among the three shallowest depths of the root zone (10, 20, and 30 cm), SWC at 20 cm had the highest correlation (*R*^2^ ≥ 0.46) with the four indicators. Similar to the characteristics of Gs and LAI, the Tc and DNAS showed the largest *R*^2^ value of 0.53 with SWC at 20 cm, while the *R*^2^ values for CTSD and CTCV were 0.47 and 0.46, respectively. The same trend was detected for SWC at the soil depth of 10 cm. However, we detected the opposite trend in the correlations between the four indicators and SWC at the depth of 30 cm, with slightly larger *R*^2^ values of 0.40 and 0.38 for CTCV and CTSD, respectively, and a smaller *R*^2^ value of 0.34 for Tc and DANS. Further research is required to determine the reason for this phenomenon.

**Table 2:** Coefficient of determination (*R*^2^) of four Tc-based crop water stress indicators with respect to the soil volumetric water content (SWC) at different depths of root zone (10, 20, 30, 45, 60, and 90 cm) on day of year (DOY) 193.

Depth (cm)	Tc	DANS	CTSD	CTCV
10	0.40*	0.40*	0.23	0.20
20	0.53*	0.53*	0.47*	0.46*
30	0.34*	0.34*	0.38*	0.40*
45	0.04	0.04	0.01	0.01
60	0.00	0.00	0.00	0.01
90	0.10	0.10	0.09	0.07

Tc, DANS, CTSD and CTCV were abbreviations of canopy temperature derived by the red–green ratio index (RGRI)-Otsu method for degrees above non-stress, standard deviation of canopy temperature, and canopy temperature coefficient of variation, respectively.

*p < 0.05.

### Mapping Maize Water Stress Based on UAV Thermal and RGB Images

**Figure 12:**
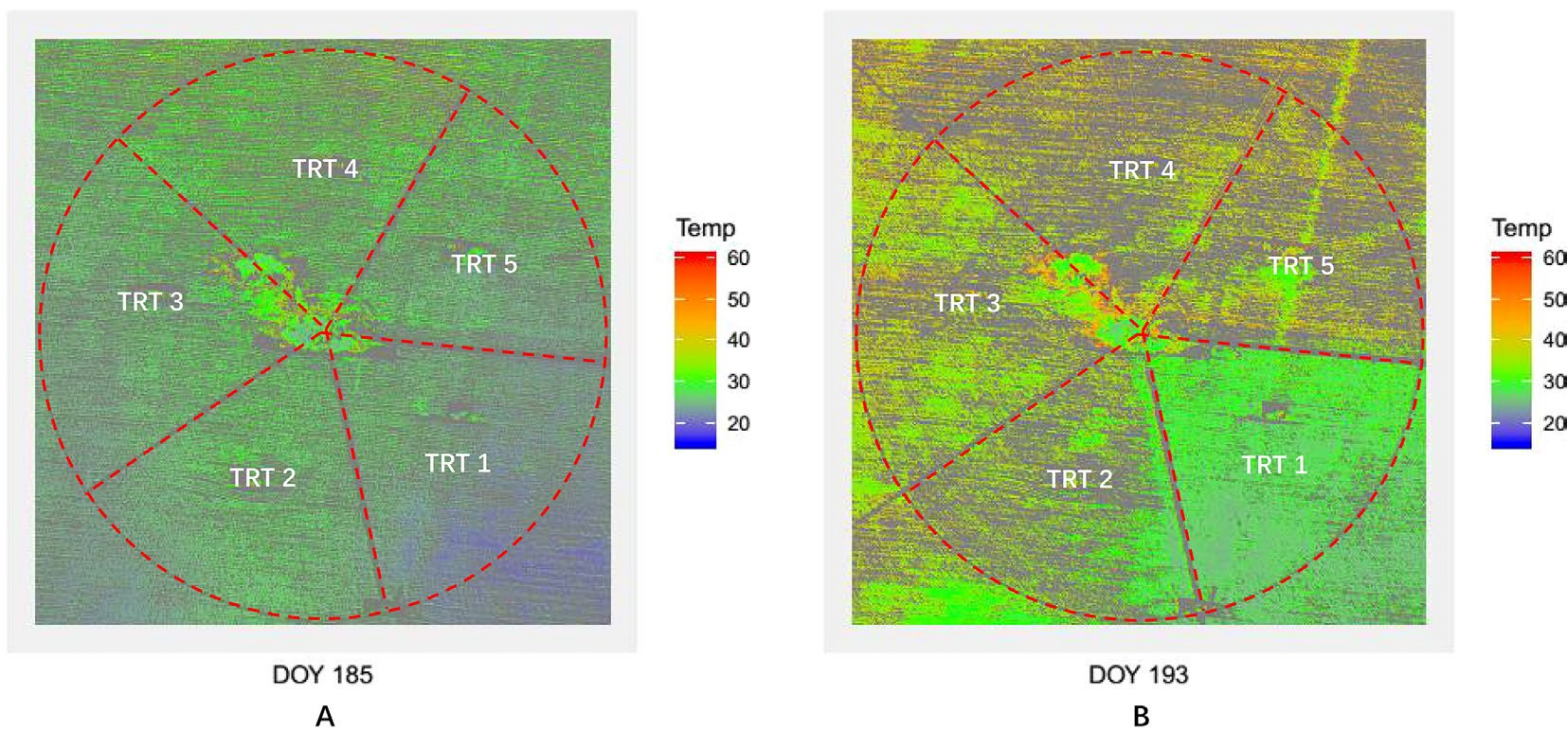
Maps of maize canopy temperature (Tc) derived by the co-registration approach (red–green ratio index (RGRI)-Otsu). Panel **(A)** for Tc maps derived by using unmanned aerial vehicle (UAV) thermal and red–green–blue (RGB) imagery acquired on day of year (DOY) 185; **(B)** for Tc maps derived by using UAV thermal and RGB imagery acquired on DOY 193.

After the comparison and analysis of the correlations between Tc-based crop water stress indicators and Gs, the classical water stress indicator, the maize Tc derived by the RGRI-Otsu method showed great potential for monitoring maize water stress. **Figure 12** shows the maize Tc distribution in TRTs 1–5 on DOY 185 and 193. On DOY 185 (non-stressed, **Figure 7C**), there were no obvious differences among three irrigation treatments (TRTs 1, 2, and 5) with mean Tc values of 25.1°C, 25.9°C, and 26.2°C, respectively (**Table 3**). However, on DOY 193 (water-stressed, **Figure 7D**), there was a clear temperature gradient among the different irrigation treatments (TRTs 1, 2, and 5) with mean Tc values of 27.1°C, 33.2°C, and 38.9°C, respectively.

**Table 3:** The mean maize canopy temperature of each deficit irrigation treatment (TRTs 1–5) derived by the co-registration approach (red–green ratio index (RGRI)-Otsu).

Dates	The mean maize canopy temperature (룗°C)				
	TRT 1	TRT 2	TRT 3	TRT 4	TRT 5
DOY 185	25.1	25.9	26.2	28.5	26.2
DOY 193	27.1	33.2	37.4	40.1	38.9

## Discussion

Thermal remote sensing using UAVs has great potential for the detection and monitoring of drought stress and has been used to monitor drought stress in crops such as cotton (Cohen et al., 2015; Cohen et al., 2017; Zhang et al., 2018b; Bian et al., 2019), potato (Rud et al., 2014), and soybean (Maimaitijiang et al., 2017; Bai and Purcell, 2018), and in orchards (Berni et al., 2009; Park et al., 2017) and vineyards (Espinoza et al., 2017; Poblete et al., 2018). However, accurately extracting the crop Tc is still a challenge (Vadivambal and Jayas, 2011; Gago et al., 2015). Because there is no thermo-electric cooler device in the uncooled thermal camera, appropriate calibration is required for accurate estimates of crop Tc. There are five main aspects of calibration: non-uniformity correction, defective pixel correction, shutter correction, radiometric calibration, and temperature calibration (for details of these factors, see Ribeiro-Gomes et al., 2017). In currently used uncooled thermal cameras, non-uniformity correction, defective pixel correction, and shutter correction are performed by the firmware included in the system. With respect to radiometric calibration, the perceived temperature of the vegetation is substantially affected by air temperature, relative humidity, emissivity, and object distance (Aubrecht et al., 2016; Sagan et al., 2019). The FLIR Vue Pro R 640 thermal camera used in this study could perform radiometric calibration *via* its digital acquisition system. For temperature calibration, a linear regression model between ground-truth temperatures measured using a handheld infrared thermometer and those derived from UAV thermal images is often established (Harvey et al., 2016; Bian et al., 2019; Sagan et al., 2019). In this study, we generated a specific linear regression model for each acquisition height (10–60 m) of the UAV thermal images (**Figure 8**), since the temperature obtained by the thermal camera significantly decreased with increasing flight height. Similar results were reported by Yang et al. (2018), who found that the linear regression model based on data acquired at 1-m height had a slope of 1.0, while that based on data acquired at 50-m height had a slope of 1.4. Similar to our results, their findings showed that the temperatures derived from thermal images acquired at 50-m height were lower than those derived from thermal images acquired at 1-m height. Therefore, to obtain an accurate temperature calibration model, specific linear regression models between ground-truth temperatures and temperatures derived from UAV thermal images should be established for different image acquisition heights.

Before the crop reaches its effective canopy cover, another problem for effectively monitoring water status based on UAV thermal imagery is the relatively low spatial resolution (image resolution ranging from 320 × 240 to 640 × 480 pixels) (Gago et al., 2015). Mixed pixels consist of crop canopy and background and considerably reduce data quality (Jones and Sirault, 2014). In this study, the RGRI-Otsu method was used to extract maize Tc at the late vegetative stage using both UAV thermal and RGB images. In the RGRI-Otsu method, high-spatial-resolution (1.25 cm) UAV RGB images were used to obtain the distribution of maize (FVC map), and the nearest-neighbor algorithm was applied to resample the spatial resolution of FVC map to match the scale of thermal images (from 1.25 to 7.8 cm). During resampling, the nearest-neighbor interpolation set the pixel value of each point of the target image to the nearest point in the source image without producing mixed pixels. This decreased the effect of mixed pixels on Tc extraction.

The RGRI-Otsu extracted Tc was highly correlated with the ground-truth Tc, with *R*^2^ of 0.94 (*n* = 15) and RMSE of 0.7°C (**Figure 9**). We detected significant deviation from the 1:1 line with the slope and intercept of 0.71 and 9.53°C, respectively. Similar deviations have been found in other studies (Baluja et al., 2012; Yang et al., 2018; Sagan et al., 2019). More specifically, in this study, when Tc was lower than 32.9°C, a lower RGRI-Otsu extracted Tc value was obtained, and when Tc was higher than 32.9°C, a higher extracted Tc value was obtained. This phenomenon is illustrated in **Figure 12** and **Table 3**. For example, on DOY 185, since maize was not under water stress (**Figure 7C**), there were relatively low mean Tc values of 25.1°C, 25.9°C, and 26.2°C for TRTs 1, 2, and 5, respectively, compared with the ground-truth Tc values of 29.2°C, 28.9°C, and 28.7°C, respectively. On DOY 193, after prolonged water deficit, TRT 2 and TRT 5 were under different levels of water stress with average Gs values of 0.24 and 0.17 mol·m^−2^·s^−1^, respectively (**Figure 7D**). The Tc also had a clear gradient with mean values of 27.1°C, 33.2°C, and 38.9°C for TRTs 1, 2, and 5, respectively (**Table 3**). Compared with the ground-truth Tc values of 29.4°C and 35.3°C for TRT 1 and TRT 5, respectively, the Tc of TRT 1 extracted by the RGRI-Otsu method (29.4°C, less than 32.9°C) was assigned a lower value of 27.1°C and that of TRT 5 (35.3°C, greater than 32.9°C) was assigned a higher value of 38.9°C. However, in TRT 2, there was no obvious difference between the ground-truth Tc value (32.7°C, close to 32.9°C) and the Tc value (33.2°C) derived by RGRI-Otsu method. Similar results were found by Ribeiro-Gomes et al. (2017). In their study, compared with the ground-truth temperature of a vineyard obtained using a FLIR B660 thermal camera, temperature derived from a UAV thermal image taken 1 day after irrigation was lower, and temperature derived from a UAV thermal image taken 7 days after irrigation was higher.

A possible reason for the phenomenon described above may be differences in maize LAI caused by drought stress. When we extracted the Tc of TRT 1 (lower than 32.9°C, **Figure 7B**) using the RGRI-Otsu method, the leaves of maize were not curled because there was no water stress (Gs > 0.50 mol·m^−2^·s^−1^, **Figure 7D**), resulting in a greater proportion of shadowed soil and leaves (lower temperature) in the UAV thermal orthophoto. When Tc was extracted from thermal images, the Tc values were lower because of the influence of shadowed soil and leaves. As water stress became more severe, the maize leaves gradually curled, resulting in a gradual decrease in the proportion of shadowed soil and leaves, and a gradual increase in the proportion of sunlit soil (higher temperature). When Tc was extracted from thermal images, the Tc values were higher due to the influence of sunlit soil. Therefore, to obtain more accurate values for the maize Tc, more accurate methods for canopy detection and extraction and better strategies for eliminating mixed pixels are needed, especially for drought-stressed maize plants with curled leaves and, thus, narrower blade width.

After Tc was extracted, selecting an appropriate Tc-based water stress indicator is an important step in effectively monitoring crop water stress status. In this study, Tc, DANS, CTSD, and CTCV, which only require Tc, were chosen to reduce the number of parameters required to detect crop water stress. Maize leaf Gs was used as the reference for water stress status. We analyzed the relationships between the four indicators and Gs, and we found that all of them could be used to monitor maize water stress with *R*^2^ values greater than 0.54 (**Figure 10**). Compared with CTCV and CTSD, Tc and DANS were more effective indicators of water stress in maize and were better able to reflect the status of SWC at shallow root zone depths.

When monitoring crop water stress over a longer period (e.g., the whole growing season), the effects of meteorological conditions on the stability of the monitoring performance of the four indicators should be normalized (Cohen et al., 2015; Cohen et al., 2017). Even the widely used CWSI empirical model is affected by different meteorological conditions. Some researchers have reported that the non-water-stress baseline of the CWSI empirical model varies markedly among different locations (Idso et al., 1981; Gardner et al., 1993; Yazar et al., 1999; Han et al., 2018) and that differences in meteorological conditions (e.g., radiation and wind speed) are among the most important factors (Zolnier et al., 2001; Payero et al., 2005; Payero and Irmak, 2006; Gonzalez-Dugo et al., 2014). For instance, the previously reported slopes of maize non-water-stress baselines established at different locations range from −1.10 to −3.77°C/kPa, and the corresponding intercepts range from 0.42°C to 3.11°C. Even in the same growing season, the coefficient of the maize non-water-stress baseline can vary significantly among different growing stages (Zhang et al., 2019). Therefore, further research is needed to compare the performance of the four Tc-based water stress indicators under different meteorological conditions.

Finally, Tc maps at the farm scale were obtained using both UAV thermal and RGB images (**Figure 12**). The Tc extracted by the RGRI-Otsu method could effectively monitor maize water stress and its spatial variability at the late vegetative stage. In addition, whether there was water stress or not, TRT 4 (moderate deficit irrigation) had the largest Tc values of 28.5°C on DOY 185 and of 40.1°C on DOY 193. Further research is required to determine the reason for this result.

## Conclusions

Before a crop reaches effective canopy cover, the accurate extraction of Tc from UAV-based thermal imagery is still a challenge. To improve the accuracy of Tc extraction, we explored methods for appropriate temperature calibration and reduction of the influence of mixed pixels on the accuracy of the extracted Tc. The number of parameters required for crop water stress monitoring and the difficulty in obtaining measurements are among the current limitations. To determine the effects of flight height on the temperature calibration of UAV thermal imagery, we conducted regression analyses between the temperatures derived from UAV thermal images acquired at different flight heights (10–60 m) and ground-truth measurements. To reduce the influence of mixed pixels on the quality of the extracted Tc value, we propose the use of the RGRI-Otsu method, which uses both UAV thermal and RGB images. Four crop water stress indicators were tested including Tc, DANS, CTSD, and CTCV. All of these indicators only need Tc. Our results confirmed that there was a specific temperature calibration model for each acquisition height (10–60 m) of UAV thermal images, since the temperature obtained by the thermal camera significantly decreased as the flight height increased. The Tc extracted by the RGRI-Otsu method was highly correlated with the ground-truth measurements with *R*^2^ of 0.94 (*n* = 15) and RMSE of 0.7°C. At the same time, there was a significant deviation from the 1:1 line with a slope and intercept of 0.71 and 9.53°C, respectively. The change in maize LAI caused by water stress (i.e., leaf curling) might explain this phenomenon. The four Tc-based crop water stress indicators all showed high correlations with Gs (*R*^2^ > 0.54), suggesting that the RGRI-Otsu method based on the combination of UAV RGB and thermal images has great potential for monitoring water stress in maize crops.

## Data Availability Statement

The datasets generated for this study are available on request to the corresponding author

## Author Contributions

LZ, YN, and WH conceived and designed the experiments; LZ and YN proposed the method and analyzed the data; LZ, YN, GL, JT, and XP discussed and drafted the manuscript. HZ and WH revised the manuscript and edited English language. All authors read and approved the final version.

## Funding

This study was supported by the 13th Five-Year Plan for Chinese National Key R&D Project (2017YFC0403203), Major Project of Industry–Education–Research Cooperative Innovation in Yangling Demonstration Zone in China (2018CXY-23), and the 111 Project (No. B12007).

## Conflict of Interest

The authors declare that the research was conducted in the absence of any commercial or financial relationships that could be construed as a potential conflict of interest.

## Abbreviations

UAV, unmanned aerial vehicle; Tc, canopy temperature; LAI, leaf area index; Gs, stomatal conductance; *R*^2^, coefficient of determination; CWSI, crop water stress index; DANS, degrees above non-stress; CTSD, standard deviation of canopy temperature; CTCV, canopy temperature coefficient of variation; TRT, treatment; DOY, day of year; FI, full irrigation; MDI, moderate deficit irrigation; SDI, severe deficit irrigation; SWC, soil water content; FVC, fractional vegetation cover; RGRI, red–green ratio index.
